# A Briefly Argued Case That Asgard Archaea Are Part of the Eukaryote Tree

**DOI:** 10.3389/fmicb.2018.01896

**Published:** 2018-08-15

**Authors:** Gregory P. Fournier, Anthony M. Poole

**Affiliations:** ^1^Department of Earth, Atmospheric and Planetary Sciences, Massachusetts Institute of Technology, Cambridge, MA, United States; ^2^Bioinformatics Institute, Te Ao Mārama – Centre for Fundamental Inquiry, School of Biological Sciences, The University of Auckland, Auckland, New Zealand

**Keywords:** Asgard, Archaea, Eukarya, eukaryogenesis, cladistics, systematics, synapomorphy, Domains

## Abstract

The recent discovery of the Lokiarchaeota and other members of the Asgard superphylum suggests that closer analysis of the cell biology and evolution of these groups may help shed light on the origin of the eukaryote cell. Asgard lineages often appear in molecular phylogenies as closely related to eukaryotes, and possess “Eukaryote Signature Proteins” coded by genes previously thought to be unique to eukaryotes. This phylogenetic affinity to eukaryotes has been widely interpreted as indicating that Asgard lineages are “eukaryote-like archaea,” with eukaryotes evolving from within a paraphyletic Archaea. Guided by the established principles of systematics, we examine the potential implications of the monophyly of Asgard lineages and Eukarya. We show that a helpful parallel case is that of Synapsida, a group that includes modern mammals and their more “reptile-like” ancestors, united by shared derived characters that evolved in their common ancestor. While this group contains extinct members that share many similarities with modern reptiles and their extinct relatives, they are evolutionarily distinct from Sauropsida, the group which includes modern birds, reptiles, and all other amniotes. Similarly, Asgard lineages and eukaryotes are united by shared derived characters to the exclusion of all other groups. Consequently, the Asgard group is not only highly informative for our understanding of eukaryogenesis, but may be better understood as being early diverging members of a broader group including eukaryotes, for which we propose the name “Eukaryomorpha.” Significantly, this means that the relationship between Eukarya and Asgard lineages cannot, on its own, resolve the debate over 2 vs. 3 Domains of life; instead, resolving this debate depends upon identifying the root of Archaea with respect to Bacteria.

## Introduction

The discovery of Archaea (Woese and Fox, 1977) has had a transformational impact on biology. Greatly expanding our knowledge of biological diversity, it became apparent that all life on earth is grouped within three primary Domains, the Archaea, Bacteria, and the Eukarya (Woese et al., 1990), with the root of the tree of life subsequently being placed between Bacteria and the lineage leading to Archaea and Eukarya (Gogarten et al., 1989; Iwabe et al., 1989). However, shortly following their discovery, alternative phylogenetic analyses proposed that the Archaea are not monophyletic (Rivera and Lake, 1992), and a stream of more recent analyses have come to a similar conclusion using a variety of expanded datasets and evolutionary models (Gribaldo et al., 2010; Williams et al., 2013, 2017; Raymann et al., 2015). These results suggest that eukaryotes evolved from within the diversity of extant Archaea, and imply that Eukarya should not be considered a Domain of equal primacy to Archaea (Gribaldo et al., 2010; Williams et al., 2013). However, the specific relationships between major archaeal groups, as well as between Archaea and eukaryotes, are often inconsistent and/or unresolved in these analyses, which are sensitive to model choice, taxon sampling, and choice of aligned sequences (Lasek-Nesselquist and Gogarten, 2013; Raymann et al., 2015; Nasir et al., 2016).

There is no doubt that the eukaryotes descend from a common ancestor (the Last Eukaryotic Common Ancestor, or LECA), as there are many characters that trace back to LECA, uniting modern eukaryotes as a group. These characters include the mitochondrion, nuclear envelope, nuclear pores and an endomembrane system, linear chromosomes, spliceosomal machinery and introns, among many others (reviewed in Koonin, 2010; Koumandou et al., 2013). Many of these traits are associated with genes that appear to be specific to eukaryotes (Hartman and Fedorov, 2002), suggesting that both the traits and the genes underlying them were acquired in the stem lineage separating eukaryotes from Archaea and Bacteria (Poole, 2010). However, recent metagenomics sampling studies from a range of environments have revealed the existence of multiple lineages (dubbed Asgard) which have many archaeal-type genetic and physiological characters but which are most closely related to eukaryotes in molecular phylogenies. Taken together, these lineages, although still only known from metagenomic sequences, significantly expanded known prokaryote diversity, challenging and requiring a re-evaluation of the evolutionary relationships between groups of Archaea and eukaryotes. Asgard lineages have also been found to possess genes encoding “eukaryote signature proteins” (ESPs) (Spang et al., 2015; Klinger et al., 2016; Zaremba-Niedzwiedzka et al., 2017), i.e., genes coding for proteins once thought to be specific to eukaryotes (Hartman and Fedorov, 2002).

The prospect of “eukaryote-like Archaea” carrying features or genes previously thought to be diagnostic of eukaryotes raises a complex and intriguing problem. How does one define the difference between Eukarya and Archaea, and how does formalizing this distinction inform hypotheses of eukaryogenesis? Drawing parallels with the evolution of mammals and birds from their reptilian forebears, we show that this dilemma can be readily navigated using an established systematics framework. Doing so enables us to separate out the evolutionary significance of the Asgard lineages from the 2- vs. 3-Domains debate, and indicates that, given current data, Asgard lineages should be considered an early offshoot of the eukaryote lineage. As such, these together represent a clade of undetermined taxonomic rank, depending upon the internal topology and rooting of Archaea.

## Results

### “Eukaryote-Specific” Proteins Encoded in Asgard Lineages

Asgard lineages carry a majority of features that are clearly associated with Archaea (Spang et al., 2015; Zaremba-Niedzwiedzka et al., 2017). However, on many molecular phylogenies, they appear as a sister group to eukaryotes (Eme et al., 2017), and are distinct from other archaea in that they carry many ESP genes associated with processes hitherto known only from eukaryotes. The list is extensive, but includes putative homologs of genes known to be involved in the cytoskeleton, vesicular trafficking, and endosomal sorting, nucleocytoplasmic transport, and eukaryote-like ubiquitinylation (Klinger et al., 2016; Eme et al., 2017; Hennell James et al., 2017). As these processes are hallmark features of eukaryotes traceable to the LECA (Koumandou et al., 2013), their presence within Asgard lineages is strongly supportive of the hypothesis that they have a closer affinity to eukaryotes than do other lineages within the Archaea (Dey et al., 2016; Eme et al., 2017).

To address the question of how we should treat Asgard lineages, it is first necessary to frame the ongoing debates concerning the evolutionary relationships between Archaea and Eukarya. As noted in the introduction, there are two competing phylogenetic interpretations; in the 3-Domain tree, Archaea, Eukarya, and Bacteria are each monophyletic. By contrast, 2-Domain trees describe phylogenies with different topologies that are united by the fact that they fail to recover the monophyly of Archaea. In order to explore the significance of the Asgard lineages, we investigate the scenario under which the proposed sister relationship between eukaryotes and Asgard has been correctly identified. It should be noted that this scenario is not universally accepted; some analyses have favored a monophyletic Archaea including Asgard lineages but excluding Eukarya, showing the recovered close relationship between Lokiarchaeota and Eukarya to be highly sensitive to the inclusion of fast-evolving archaeal lineages, and protein dataset selection (Da Cunha et al., 2018). However, the impact of these biases continues to be debated (Spang et al., 2018), and phylogenetic analyses of ESP genes within Eukarya and Asgard generally support a close relationship (Spang et al., 2015). The most parsimonious evolutionary scenario would have these gene trees congruent with a species tree in which Eukarya and Asgard share a most recent common ancestor. These gene distributions can also be reconciled with other species tree topologies and/or patterns of character acquisition, such as an earlier origin of ESPs with losses in other archaeal lineages (Forterre, 2013), or ancient HGT of genes encoding ESPs. While not parsimonious, these alternative hypotheses cannot yet be summarily excluded. These issues are not independent from the 2- vs. 3-Domains question, as well as the rooting of the archaeal tree. The topology of the broader tree is important, and we shall return to this.

### How Systematics Deals With Characters Guides How We Should Deal With ESPs

As the name implies, ESP genes were previously thought to encode eukaryote-specific proteins. However, if they are found outside the Eukarya, this is potentially a misnomer. Some ESPs are not eukaryote-specific at all, and we are only now recognizing that these are more widespread than previously thought. Indeed, as neatly summarized in a recent review by Eme et al. (2017), some appear to be distributed across eukaryotes, Asgard and TACK lineages of Archaea. With this broader sampling of archaeal diversity, we can also discern a pattern, where ESPs shared by TACK lineages and Asgard tend to be informational and ribosomal proteins that are related to highly conserved cellular functions across life, while ESPs found extensively across Asgard lineages are closely associated with specific eukaryal cytological structures and processes (cytoskeleton and vesicle trafficking). These latter ESPs are absent within TACK (except for the ESCRT-III protein, a distant homolog of actin, and a distant homolog of tubulin specifically within Thaumarchaeota). This large cohort of ESPs that are shared by Eukarya and Asgard to the exclusion of other archaeal lineages is therefore distinctly relevant to the early evolution of eukaryote-like cells, and suggests that the long evolutionary grade leading to crown group Eukarya includes Asgard, to the exclusion of other archaeal lineages, including TACK.

What is the cladistics significance of ESPs in Asgard lineages? Systematics deals with this issue, through its definition of derived characters. Within a phylogenetic tree, clades are defined as natural, monophyletic groups that possess shared derived characters, that is, characters that were acquired along the stem lineage leading to the group, and as such are absent from other lineages (Hennig, 1966). Such characters are termed *synapomorphies*, and are the foundation of modern systematics in evolutionary biology. Only synapomorphies are appropriate for defining clades, but not all shared characters are synapomorphies. If a character is shared by an outgroup as well, it is considered a shared ancestral character, or *symplesiomorphy*. Symplesiomorphies are not appropriate characters for defining a natural group (Hennig, 1966).

Applying these cladistics principles to our two classes of “ESPs” is straightforward. Those which have been found to be widespread (present in Eukarya plus many Archaea) are symplesiomorphies, having evolved well before eukaryotes. We contend that ESPs that are shared with Asgard lineages, but not with other Archaea, are best interpreted as synapomorphies if the sister relationship between Asgard and Eukarya is correct, and that these together form a natural monophyletic group to the exclusion of other Archaea. Importantly, this does not make Asgard lineages eukaryotes; there is no evidence (as yet) that these lineages carry nuclei or other endomembrane systems associated with the eukaryote cell. Moreover, it does not indicate that Asgard lineages are *not* archaeal; under 2-Domain tree topologies, both Asgard and eukaryotes are archaeal in systematic terms, regardless of their sister relationship or any synapomorphies acquired within stem eukaryotes.

### An Analogy From Vertebrate Paleontology

**FIGURE 1:**
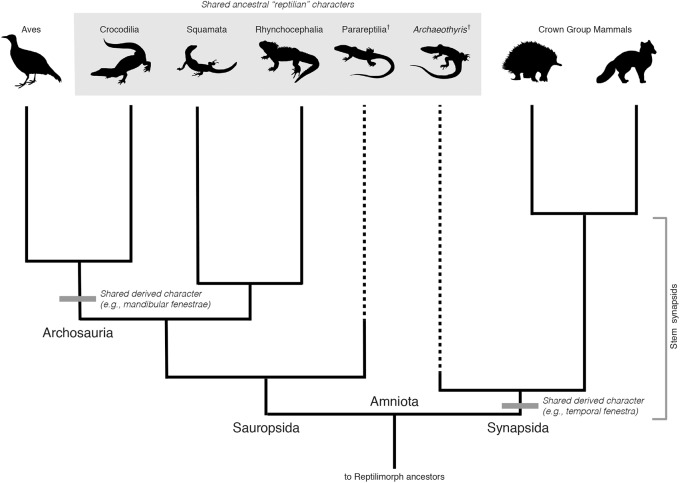
Phylogeny of selected major groups of amniotes. Selected synapomorphies are indicated by gray bars. Two basal extinct lineages Parareptilia and *Archaeothyris* (representing Ophiacodontidae) are included to indicate the retention of shared ancestral characters (large gray box). Sauropsida includes all extant reptiles and their extinct relatives. Extant anapsids (turtles) are omitted due to their uncertain placement within Sauropsida. Synapsida and Sauropsida are clades of equal taxonomic rank, with a last common ancestor congruent with the ancestor of all crown group amniotes (Amniota). Archosauria is a clade nested within Sauropsida. As such, it has a lower taxonomic rank than either Sauropsida or Synapsida, even if shared derived characters of this group distinguish them from other amniotes. Representative taxon images were downloaded from PhyloPic (http://phylopic.org/). All images are uncopyrighted except parareptilia (http://phylopic.org/image/00b96cf3-1802-4bda-a6cc-76aea0f6f05e/) which is owned by Nobu Tamura (vectorized by T. Michael Keesey), under the following creative commons license (https://creativecommons.org/licenses/by-sa/3.0/legalcode).

To better illustrate the nature of these relationships, it is helpful to consider a better-understood case, the evolutionary relationship between mammals and reptiles (**Figure 1**). The deepest split in the evolutionary history of amniotes is that between the groups Sauropsida and Synapsida, congruent to the split between extant reptiles and mammals. After this deep divergence, Sauropsida diversified into groups including all extant and extinct reptile lineages, including birds. Synapsida diversified into many lineages as well. Of these, only mammals survived, although there is a rich, diverse evolutionary history of extinct mammalian relatives and ancestors (Benton, 2015). During synapsid evolution, all of the synapomorphies present within extant mammals were acquired over ∼160 million years, with the evidence of these accumulated changes preserved within the geological record as fossils representing members of *stem groups*. The earliest known representatives of the stem mammal lineage are almost entirely “reptilian” in their morphology, reflecting the retention of many ancestral characters or symplesiomorphies from their early amniote ancestors (Benton, 2015). For example, one of the earliest known synapsids from the late Carboniferous (∼306 MYA), *Archaeothyris florensis*, shares a large number of morphological characters with members of Sauropsida, and was “lizard-like” in appearance (Reisz, 1972; Falcon-Lang et al., 2007; Benson, 2012; **Figure 1**). Nevertheless, the presence of some shared derived characters, including a single temporal fenestra and slightly enlarged canines identify this extinct group as members of Synapsida (Kammerer et al., 2014), more closely related to mammals than extant reptiles or birds. Strictly speaking, *A. florensis* was not a reptile.

If archaeal-like characters were ancestral to both eukaryotes and Archaea (as is likely), one would expect the earliest relatives of the ancestors of modern eukaryotes to resemble Archaea, with perhaps only a few derived characters in common with extant eukaryotes. In such a case, while the majority of characters would be ancestral and archaeal-like, under a systematics framework and a clade-based definition of Archaea, these characters (symplesiomorphies) do not, on their own, bestow status as Archaea to the exclusion of Eukarya. Rather, being more closely related to Eukarya than the archaeal outgroup, and being united by derived characters shared with Eukarya, they would be properly identified as belonging to a clade including Eukarya. Unlike *A. florensis* and its relatives, representatives of such early forms may have survived as the Asgard lineages.

### Applying the Principles of Cladistics to Eukarya, Asgard, and Archaea

The phylogenetic placement of eukaryotes with relation to Archaea is a different question than the process of the origin of the eukaryote-type cell. These questions are related by the eukaryal stem, the lineage along which all eukaryote-specific characters were acquired during perhaps over a billion years of evolution. The deeper the stem ancestor, the fewer derived characters will be present. It directly follows that, immediately following the divergence of the eukaryal stem lineage from its outgroup, one would expect few, if any derived characters, and the preservation of most, if not all ancestral characters. An abundance of ancestral characters present within a deeply branching lineage is thus a poor rationale for its placement with the outgroup, rather than with a more highly derived sister group. This observation is important in establishing principles for accurately describing the ancestry of eukaryotes.

Using a clade-based approach, defining eukaryotes is trivial – they are all descendants of the last common ancestor of extant Eukarya, i.e., “crown Eukarya.” All extinct stem lineage members then belong to broader groupings inclusive of crown Eukarya, based on nested subsets of synapomorphies, as is the case with increasingly mammal-like groups nested within the synapsid lineage leading to crown group mammals [although, unlike mammals, we do not have any fossil evidence of these likely extinct stem groups that indicate the order of character acquisition (Poole, 2010)]. Alternatively, the definition from a maximally inclusive stem-based approach is equally trivial, including extant Eukarya and all extinct relatives more closely related to Eukarya than any other extant group. However, neither of these schemas inform which characters are important in distinguishing Eukarya from Archaea, or in interpreting the relationship between Eukarya and other *extant* lineages more closely related to Eukarya than Archaea (such as, presumably, Asgard). Synapomorphy-based approaches (i.e., identification of shared-derived characters, such as ESPs) are necessary to provide this distinction.

**FIGURE 2:**
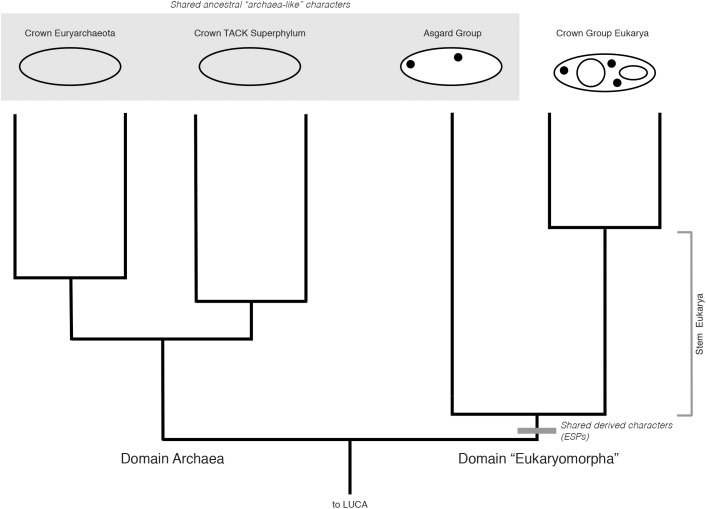
Hypothetical rooted archaeal tree including Asgard and eukaryotes, consistent with a “3-Domain” topology. ESPs (black dots) are synapomorphies uniting Asgard and eukaryotes as a clade (gray bar). Additional archaeal groups (e.g., DPANN) are omitted for clarity. Archaeal and Asgard groups share many “archaeal-like” ancestral characters (large gray box). Archaea are depicted as rooted between Euryarchaeota and TACK, although this hypothetical 3-Domain topology is also valid under all other rootings for this group. Despite these ancestral characters, with this rooting, Asgard + Eukarya together constitute a clade of equal taxonomic rank to that of Archaea. Here, we propose the name “Eukaryomorpha,” reflecting the shared characters uniting this group and distinguishing them from the archaeal outgroup.

Cladistically, therefore, the monophyly of Eukarya and Asgard does not, on its own, define Archaea as paraphyletic. Rather, a rooting between this group and Archaea (as discovered by the placement of the outgroup, Bacteria) would exclude Asgard from Archaea, and remain entirely consistent with a 3-Domain Tree of Life, with Asgard included within a broader sister clade also including Eukarya, and of equal taxonomic rank to Archaea (**Figure 2**). The analogy here is to Sauropsida and Synapsida in **Figure 1**, where reptile-like forms are present on both sides of tree, but are not diagnostic of clade membership. While this rooting has not been recovered so far by any major published phylogenetic analyses including Archaea, Asgard, and Eukarya, the rooting of the archaeal tree remains contentious, and is highly sensitive to evolutionary and phylogenetic reconstruction models, taxon sampling, protein datasets, and alignment site sampling (e.g., Lasek-Nesselquist and Gogarten, 2013; Raymann et al., 2015). The impact of these factors on the rooting of a tree including Archaea, Asgard, and Eukarya have yet to be tested. A rooting congruent with earlier 3-Domain topologies remains plausible, and the implications thereof are important to consider, especially in light of character-based arguments for the unity of Asgard and Archaea made independently of phylogenetic evidence.

One prediction of this systematic framework and rooting is that, if Asgard + Eukarya do in fact group outside of Archaea, there may also be derived, archaeal-specific characters acquired in the stem of the archaeal sister group, that is, genes (for Archaeal-specific proteins, ASPs) present across Euryarchaeota, DPANN, and TACK clades, but absent in Asgard and Eukarya. Complete genome sequencing of members of the Asgard superphylum will allow this prediction to be tested.

### Two Domains, and an Analogy for Alternative Rootings

**FIGURE 3:**
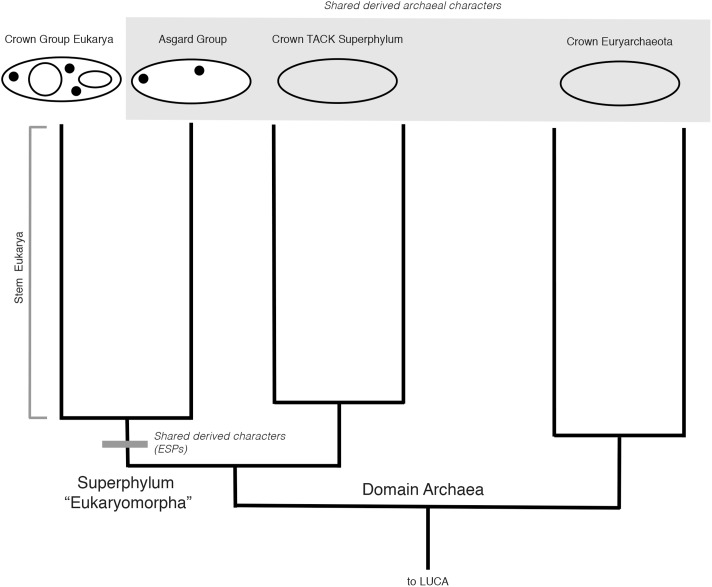
Hypothetical rooted archaeal tree including Asgard and eukaryotes, consistent with a “2-Domain” topology. With this rooting, the Asgard + Eukarya clade is nested within Archaea, i.e., the last common ancestor of all Archaea (excluding Asgard) is also the last common ancestor of Eukarya and Asgard. As such, Asgard + Eukarya constitute a clade of unequal taxonomic rank to Archaea. Note that the scenarios in **Figures 2**, **3** only differ in the placement of the root, not in the topology of the tree or mapping of the characters.

What if Archaea + Eukarya are rooted on a different branch, so that a monophyletic Eukarya + Asgard groups within extant archaeal diversity? As a consequence of this rooting, Archaea would in fact be paraphyletic, consistent with a “2-Domain” hypothesis for the Tree of Life. How then, would Eukarya’s relationship to Asgard be best understood? If Eukarya roots within extant archeal diversity, one can more clearly polarize the shared ancestral characters within Eukarya, and infer that the earliest stem eukaryotes were essentially archaeal, in both a systematic and physiological sense. However, the same shared derived characters of ESPs would still unite Eukarya and Asgard, and their monophyly would still stand, albeit as a clade of a lower taxonomic rank than Archaea (**Figure 3**).

It is worth considering another parallel case from vertebrate evolution depicted in **Figure 1**, that of the relationship between Aves (birds) and other reptilian lineages. The extant sister group to birds, Crocodilia, retains several ancestral characters in common with other reptile outgroups, but also show derived characters linking them to birds within the wider group Archosauria, including their extinct relatives, pterosaurs and non-avian dinosaurs (e.g., mandibular fenestrae) (Benton, 2008). This placement of birds is the reason that “reptiles” and “dinosaurs” are non-natural, paraphyletic groups, united by shared characters but excluding birds. Birds are excluded because of their highly derived set of characters that distinguish them morphologically, specifically, adaptations for flight. However, this is not a systematic criterion, and rather reflects a character-based distinction that is intuitively satisfying based on historical classification schemes. Extending the analogy to Archaea, in a similar case of a paraphyletic, 2-Domain tree, should Asgard still be grouped with Eukarya to the exclusion of Archaea, based on the monophyly of these groups and their associated synapomorphies? The case would rest upon which derived characters were selected to exclude Eukarya and Asgard from Archaea, rendering the latter paraphyletic. An apomorphy-based definition would, in this case, be necessary to justify such paraphyly. If the evolutionary grade leading to Eukarya traversed a continuum of physiological and genetic innovations, selecting a single derived character defining this group to the exclusion of archaeal ancestors is not only challenging, but inherently problematic. This is the case for both the broader Asgard + Eukarya group, as well as within stem proto-eukaryotes, after their divergence from Asgard. This is a general problem within taxonomy, and does not indicate that eukaryote evolution requires special consideration, or a re-evaluation of traditional cladistics principles. However, regardless of any apomorphy-based definition of Eukarya, Asgard and Eukarya would still represent a distinct clade within Archaea, defined by shared derived characters associated with the evolution of key eukaryote-like cytological features, and requiring taxonomic recognition.

### Challenging Mito-Centrism

One derived character often elevated to be the defining trait of “true” Eukarya is the presence of mitochondria, to the extent that it has been proposed that Eukarya are the product of a “merger” of a bacterial and archaeal lineage, and that this event triggered the evolution of all additional Eukarya-specific characters, such as introns, the nucleus, and endomembrane system (Martin and Koonin, 2006). Should the endosymbiosis leading to mitochondria be considered the singular event that defines eukaryogenesis, that is, defining total group “true” eukaryotes? The acquisition of the mitochondrial lineage is undoubtedly one of the most important evolutionary events in the history of life on Earth. However, mitochondria are only the “primary” eukaryal character under the very specific hypothesis that many other eukaryal characters evolved in response to it. This is a complex evolutionary narrative requiring numerous assumptions (Lynch and Marinov, 2017), and has been challenged by alternative models in which many of these same eukaryal-specific characters are required for the uptake and maintenance of the symbiont ancestors of the mitochondrial lineage (Cavalier-Smith, 2009; Martijn and Ettema, 2013; Poole and Gribaldo, 2014; Pittis and Gabaldon, 2016). As some ESP proteins found within Asgard are orthologous to proteins in eukaryotes that are associated with vesicle formation, membrane trafficking, and cytoskeletal functions (Spang et al., 2015; Zaremba-Niedzwiedzka et al., 2017), their discovery does further support evolutionary models in which these processes were ancestral to mitochondrial acquisition. Aside from these arguments, the following evolutionary thought experiments challenge the notion of a mitocentric view of eukaryal evolution, by addressing the metabolic, genetic, and cytological features of this event.

The mitochondrial acquisition represents three specific changes to the eukaryal ancestor lineage, metabolic (the acquisition of aerobic respiration), genetic (the acquisition of a large number of bacterial genes), and cytological (the maintenance of a highly derived replicating organelle). The different aspects of this single event can, conceptually, be considered individually.

If there had never been an endosymbiotic event giving rise to mitochondria, but the genes for aerobic respiration had nevertheless been acquired by horizontal gene transfer (HGT) from an alphaproteobacterial lineage, would this event, in itself, have the same weight as a defining character for Eukarya? Metabolic innovations, including aerobic respiration, often evolve via HGT across microbial lineages. The widespread distribution of aerobic respiration across the Tree of Life shows that this character would be a poor synapomorphy to define Eukarya as a distinct Domain of life. From a genetic perspective, many genes of bacterial origin were transferred as a consequence of the mitochondrial endosymbiotic event, although it is likely that many of the genes also shared between Eukarya and Bacteria were not necessarily acquired in this way, as they have evidence of different evolutionary histories (Huang, 2013). Furthermore, a large influx of genetic information from a specific donor lineage is also frequently encountered in microbial groups [e.g., Thermotogales (Zhaxybayeva et al., 2009), Aquificales (Boussau et al., 2008), and Thermoplasmatales (Ruepp et al., 2000)]. These are not generally interpreted as singular events, but as the results of biased “highways” of gene sharing (Beiko et al., 2005), continual processes that extend along the histories of these lineages. This analogy also appears to hold for the mitochondrial lineage within Eukarya, where endosymbiotic gene transfer (EGT) began after mitochondria were acquired, likely in concert with mitochondrial genome reduction, and continued after the diversification of extant eukaryal groups in a clade-specific fashion, especially in plants (Adams and Palmer, 2003; Bonen and Calixte, 2006). If the only difference between EGT and a highway of HGT is the symbiotic intermediary, it is arguable that this is not a meaningful distinction.

**FIGURE 4:**
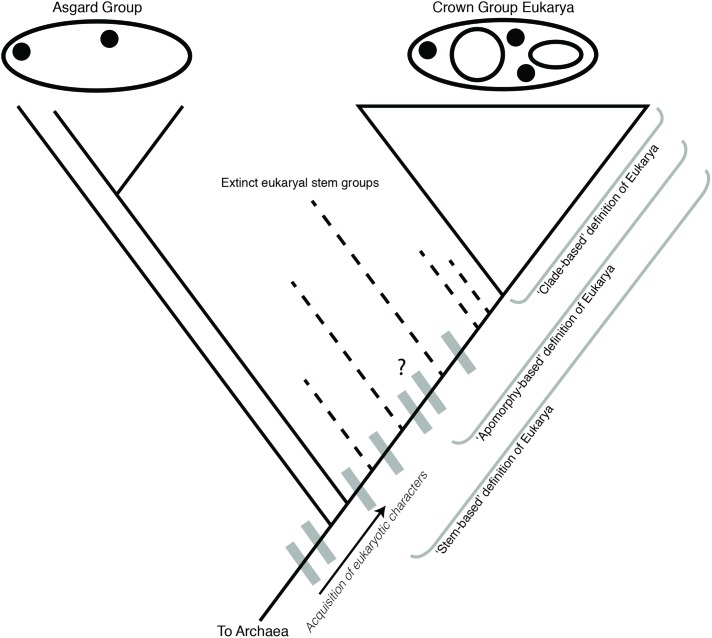
Cladogram of Asgard and eukaryote evolution as related to different definitions of Eukarya. Eukaryal-specific characters (ESPs, gray boxes) were acquired in the ancestor of Asgard + Eukarya, and continue to be acquired in the eukaryal stem lineage. An apomorphy-based definition of Eukarya requires the identification of a specific defining character for Eukarya, which would include some eukaryal stem groups, but exclude others. The selection of a defining character for an apomorphy-based definition is therefore inherently subjective. The Asgard group is depicted as an evolutionary grade, although a monophyletic Asgard group is also consistent with this model.

The acquisition of mitochondria is therefore most unique and distinct from archaeal and bacterial evolutionary processes from a cytological perspective. Obligate, co-evolved endosymbioses involving radical genome reduction are common among bacterial endosymbionts of eukaryotes (McCutcheon, 2016). In contrast with these examples, it is the intimate integration of the mitochondria with both nuclear and cytosolic cellular components and processes that argue for their being a key character of Eukarya. However, comparison with another endosymbiotic event within eukaryal evolution, the acquisition of a cyanobacterial endosymbiont establishing the plastid-containing eukaryal group Archaeplastida, provides valuable context for these claims. The evolution of eukaryal photosynthesis via plastids is at least as metabolically and physiologically significant as the evolution of aerobic respiration via mitochondria (O’Malley and Powell, 2016), and involved similarly radical rearrangements of cytological machinery and physiological innovations, including EGT and genome reduction (Nowack and Weber, 2018). Yet, none of these evolutionary events and derived characters are elevated to the degree that Archaeplastida is argued to constitute a new Domain of life arising from within a paraphyletic Eukarya. This comparison further reveals the problematic nature of a mitocentric view of eukaryal origins. Rather than focusing on this one character, it is more useful to consider stem eukaryotes as an evolutionary grade containing an ordered series of many complex derived characters, of which mitochondrial acquisition is merely one (Poole and Gribaldo, 2014). This is consistent with the interpretation of Asgard lineages being part of this grade, with ESPs shared by the Eukarya + Asgard group representing the earliest known derived characters in the process of eukaryogenesis (**Figure 4**).

## Discussion

Gene tree phylogenies tend to recover the monophyly of Eukarya and Asgard groups. ESPs have been identified in these newly discovered groups, reinforcing this proposed relationship. We propose that these results are consistent with the interpretation that Eukarya and Asgard lineages form a distinct clade defined by shared derived characters. Following the divergence of Asgard and Eukarya, stem eukaryotes continued to acquire a large number of derived physiological characters, including mitochondria, that would come to represent the “modern” eukaryotic cell. A correct application of cladistics requires grouping based on shared derived characters. Many ancestral archaeal-like characters were retained in the Asgard lineages, and these would have been present in the earliest direct stem Eukarya ancestors, as well. These ancestral characters are, in themselves, insufficient to define Asgard lineages as members of Archaea except in the sense that, under 2-Domain tree topologies, both Asgard and Eukarya are part of the archaeal tree. Therefore, a monophyletic relationship between Eukarya and Asgard cannot be used to distinguish between traditional “2-Domain” and “3-Domain” hypotheses for the Tree of Life. Rather, the placement of the root leading to Bacteria provides this distinction. While current phylogenomic analyses favor a rooting within Archaea, this placement is highly sensitive to a variety of factors, and continues to be debated. Both 2-Domain and 3-Domain scenarios are compatible with treating Asgard lineages as members of a distinct and broader group including eukaryotes that requires taxonomic recognition.

## Author Contributions

GF originated and devised the study, which was further developed in collaboration with AP. GF drafted the figures. GF and AP drafted the manuscript.

## Conflict of Interest Statement

The authors declare that the research was conducted in the absence of any commercial or financial relationships that could be construed as a potential conflict of interest.
